# Late-onset schizophrenia: a differential diagnosis

**DOI:** 10.1192/j.eurpsy.2023.2275

**Published:** 2023-07-19

**Authors:** M. V. López Rodrigo, M. Palomo Monge, A. Osca Oliver, F. Tascón Guerra, V. Ros Fons

**Affiliations:** Psiquiatría, Hospital Nuestra Señora del Prado, Talavera de la Reina, Spain

## Abstract

**Introduction:**

Regarding the diagnosis of schizophrenia, a peak of onset of symptoms is considered at 25 years. The debut after 60 years is considered late onset and is rare, generating controversies in the diagnosis

**Objectives:**

We present the case of a 58-year-old patient with no personal or family history of mental health, who came to the emergency room for the first time, reporting feeling in danger. He comments itching on his skin, verbalizing seeing bugs running through it, relating this phenomenon to “witchcraft by my brothers”, he also refers to feeling like “they watch my thoughts and block it through a mobile application, they enter through my eye right and this gives me less vision and a headache. He also refers to having the ability to listen to how his brothers talk about how they are going to “hurt me.” Psychopathologically, we highlight that she is oriented in the three spheres, presenting delusional ideation with an experience of harm, a phenomenon of thought theft and auditory and tactile hallucinations.

**Methods:**

Analytical and imaging tests, as well as toxins in urine, were negative.

**Results:**

Diagnosis of psychotic episode is made to see evolution. The clinic partially yields to treatment with atypical antipsychotics. At this time, the patient has no awareness of the disease.

**Conclusions:**

Despite being a diagnosis that is scarcely prevalent, once organic disease has been ruled out.

**Disclosure of Interest:**

None Declared

